# From chat to change: a neuroinclusive framework for cognitive empowerment and daily living skills in autism

**DOI:** 10.3389/frcha.2026.1769862

**Published:** 2026-03-31

**Authors:** Anna Meduri, Chiara Marraffa, Gaia Roccaforte, Chiara Failla, Germana Doria, Ileana Scarcella, Paola Chilà, Roberta Minutoli, Giovanni Pioggia, Flavia Marino

**Affiliations:** 1 Institute for Biomedical Research and Innovation (IRIB), National Research Council of Italy (CNR), Messina, Italy; 2 Department of Cognitive, Psychological Science and Cultural Studies, University of Messina, Messina, Italy; 3 Department of Biomedical, Dental and Morphological and Functional Imaging Sciences, University of Messina, Messina, Italy; 4 Faculty of Psychology, International Telematic University Uninettuno, Roma, Italy

**Keywords:** adaptive learning, autism spectrum disorder (ASD), biofeedback, chatbot, cognitive empowerment, daily living skills, neuroinclusive design

## Abstract

Conversational technologies are increasingly investigated as supportive tools for autistic individuals; however, existing approaches remain largely application-driven and conceptually fragmented, with limited integration between conversational design and the cognitive foundations of daily living skills. To date, no unified framework explicitly conceptualizes chatbot-based interaction as a structured environment supporting functional autonomy in autism. This Perspective article introduces the Neuroinclusive Conversational Framework (NCF), a theoretical model that reframes chatbots as cognitive scaffolds designed to support everyday functioning in addition to social simulation. Drawing on cognitive psychology, executive functioning research, mediated learning theory, and neuroinclusive design principles, the NCF conceptualizes conversational interaction as a structured learning environment capable of supporting planning, sequencing, task initiation, and self-monitoring within daily routines. The framework is articulated along three interrelated dimensions—cognitive–functional, structural–adaptive, and contextual–ecological—each addressing mechanisms relevant to cognitive accessibility, adaptability, and ecological transfer. By synthesizing evidence across diverse conversational technologies and identifying recurring design principles such as predictability, low social pressure, and adaptive structure, the NCF provides a coherent lens for interpreting existing systems and guiding future design. The article further discusses emerging directions, including biofeedback-informed adaptivity, perceptually accessible interface design, and context-sensitive interaction, emphasizing the importance of ecological validity and integration with human guidance. Overall, the NCF offers a theoretical foundation to support systematic research and development of conversational technologies aimed at strengthening daily living skills and functional autonomy in autistic individuals.

## 1 Introduction

Autism is a heterogeneous condition that affects communicative, cognitive, and adaptive functioning, with significant implications for daily activities and personal autonomy (1). In parallel with the rapid growth of conversational technologies, interest in chatbot-based supports for autistic individuals is increasing. Several established traditions already conceptualize technology as support for everyday functioning, including cognitive aids and cognitive prosthetics, executive-function supports, mediated learning and scaffolding approaches, and participation-oriented assistive and AAC perspectives. However, the field still lacks a chatbot-specific framework that links the structure of artificial dialogue to the cognitive mechanisms underpinning autonomy and daily living skills in autism.

Despite the availability of structured interventions, generalization to everyday contexts remains a persistent challenge (2). At the same time, the increasing use of digital technologies creates opportunities to design supports that accompany individuals across routines, expanding possibilities for learning and participation (3). Chatbots may contribute to this goal by providing structured and predictable interaction that supports learning and task management, complementing rather than replacing human guidance (4, 5). Evidence suggests that low-pressure and predictable conversational environments can enhance engagement and reduce communicative anxiety, making interaction more accessible for neurodivergent users (6) while systems such as LANA and Friendy illustrate how multisensory personalization and affective scaffolding can facilitate concept acquisition and continuity during intervention (7–9). Yet, current work remains distributed across isolated applications and outcome targets, without an integrative account of how conversational design choices translate into autonomy-relevant cognitive support in daily living. The Neuroinclusive Conversational Framework (NCF) addresses this gap by conceptualizing artificial conversation as a structured socio-cognitive learning environment, in which communicative interaction serves as a mechanism for cognitive scaffolding and functional empowerment. In this view, conversational design can be oriented toward autonomy-relevant processes such as memory, planning, sequencing, task initiation, and monitoring. While many applications emphasize communication or social interaction, less attention has been devoted to how conversational tools can be intentionally designed to scaffold autonomy-related cognitive processes that predict real-world independence (10). This Perspective article therefore outlines (i) a synthesis of evidence across eight descriptive macro-areas of conversational technologies in autism (Table 1), (ii) the NCF and its core axes, and (iii) implications for design, empirical testing, and future clinical translation.

**Table 1:** Eight descriptive (non-exhaustive) macro-areas of conversational technologies in autism, grouped by primary function and use context, with representative systems, modalities and reported outcomes.

Macro-area	Representative systems	Primary goal	Modality/context	Key evidence
Educational conversational tutors	LANA—Arabic CITS (7)	Learning support (school-related content)	Text-based tutoring dialogue; educational setting	CITS architecture adapted to VAK styles; emphasis on structured, personalized instruction
Therapeutic companion/clinical support chatbots	Friendy (8)	Improve cooperation/continuity during sessions; support clinicians	Context-aware text chatbot; clinical/psychotherapy setting	Modular framework; technical results + clinical feedback; positioned as scalable complement (vs. robotics)
Mental health & affective scaffolding	Contextual chatbot for depression prevention in ASD (9) Therapist Vibe (11) Alexa as Coach (12)	Emotion/anxiety support and preventive mental health scaffolding; guided reflective practice	Chatbot/smart speaker; guided exercises (e.g., CBT-informed prompts, storytelling)	Shows how structured dialogue can deliver cognitive–affective scaffolding (including preventive support), even when outcomes are framed as engagement/satisfaction
Home/IoT and smart-speaker supports	ASPECT (13) Google Assistant in therapy for children with NDD (14)	Smart-speaker–mediated supports for structured practice and/or *in-situ* prompting (e.g., speech practice, therapy routines)	Alexa/Google Home; smart-speaker interaction in home and/or therapy settings	ASPECT: scoring comparable to therapist; highlights ASR/microphone constraints. Google Assistant: identifies opportunities and barriers for clinical workflow integration (reliability, usability, facilitation needs)
Speech–language therapy supports and clinical deployment constraints	Echo as speech recognition device (15)	Speech–language practice support and assessment (e.g., echoic responding), with attention to feasibility constraints for clinical use.	Voice in/out (smart speaker); clinical workflow integration; vocabulary customization and ASR reliability as key constraints	Highlights feasibility limits (recognition accuracy, customization burden) and the need for predictable, reliable speech interaction in therapeutic deployment
Daily living, empowerment & participation	VCA for adolescents with ASD (16) Accessibility Came by Accident (17) Social Fringe Dwellers (6) General Chinese chatbot for children with ASD (18)	Daily autonomy, self-care, participation; social resilience (e.g., bullying)	Voice-based agent/IPA; everyday use + co-design; conversational modules for situated support	Shifts focus from “interaction” to ecological functions (routines, decision support, social resilience); highlights accessibility/discoverability and appropriation of mainstream tools
AAC & multimodal communication supports	Alex (6)	Communication support for non-verbal children; customization	Android app with symbols/images + conversational agent	Emphasizes customization, interoperability, and the role of therapists in system training
Screening/assessment chatbots	Aquabot (19)	Pre-screening/triage and severity estimation	Text chatbot with NLP + classification	Example of structured decision support; useful to discuss validation limits and interpretability risks

A recurring limitation across existing systems is the limited ecological transfer of learned skills. Most tools remain confined to controlled, non-naturalistic contexts and rarely address real-world routines.

## 2 Conversational technologies and autism: evidence and insights

A scoping review of the literature on conversational technologies for autism reveals a highly heterogeneous field in which systems differ widely in purpose, structure, and interaction modalities (20). To improve conceptual clarity, we grouped systems into eight descriptive macro-areas based on their primary function and use context: educational support, psychotherapy/clinical companions, mental health and affective scaffolding, speech–language therapy supports, home/IoT and smart-speaker supports, daily living empowerment and participation, AAC-oriented multimodal support, and screening/assessment. We emphasize that this taxonomy is descriptive rather than exhaustive, and that additional representative systems exist within each area. Despite variability across applications, this categorization helps summarize the state of the art and highlights shared properties. Across studies, conversational systems tend to offer predictable interaction patterns, reduced social pressure, and opportunities for structured, repeatable practice—features that align closely with the learning profiles and cognitive needs of many autistic individuals.

### 2.1 Educational conversational tutors

A first group of studies concerns educational conversational tutors. The LANA system—an Arabic Intelligent Tutoring System for high-functioning autistic children—adapts questions and explanations to the learner's perceptual preferences and demonstrates how structured dialogue can support comprehension and engagement (7). Findings from this line of work suggest that predictable conversational scaffolding may provide a cognitively accessible environment for guided learning.

### 2.2 Therapeutic companion chatbots

A second cluster of research focuses on therapeutic companion chatbots. Friendy, for example, integrates affective modelling and machine learning to assist children during psychotherapy, particularly when non-cooperation or attentional fluctuations occur. Initial evaluations indicate that such a system can help stabilize interaction and maintain engagement without replacing the clinician's role, but rather complementing it (8, 9).

### 2.3 Mental health and affective scaffolding

Beyond in-session support, a related line of work frames conversational agents as tools for emotion-focused scaffolding and preventive mental health support, using structured prompts and adaptive dialogue to guide reflection and coping. These systems further illustrate how conversational structure can function as a cognitive-affective scaffold rather than a purely social interface (8, 9).

### 2.4 Voice assistants and IoT-based systems

Parallel research investigates voice assistants and Internet of Things-based systems. ASPECT, a program built on Amazon Alexa, showed therapist-level accuracy in recognizing and modelling echoic productions, while offering children opportunities to practice vocalization within predictable, low-pressure home environments (13). Additional work on smart speakers highlights the potential value of consistency and error tolerance for structured practice, while also emphasizing practical constraints related to automatic speech recognition accuracy and feasibility (13, 15).

### 2.5 Speech–language therapy supports and clinical deployment constraints

Related work has also examined how smart speakers can be integrated into therapy workflows through speech recognition and structured prompting. These studies also highlight practical limitations, including recognition accuracy, the need for vocabulary customization, and feasibility constraints for clinical use (13, 15).

### 2.6 Daily living empowerment and participation

Importantly, a growing set of contributions focuses on everyday autonomy and participation—examining how voice-based conversational agents can support routines, self-care, and emotion regulation in naturalistic contexts, often through participatory design approaches (16). Complementary evidence from disability-focused IPA research further underscores that real-world benefits depend on accessibility, discoverability, and the match between conversational functionality and users’ routine needs (17).

### 2.7 Multimodal conversational systems and AAC-oriented chatbots

A further body of work examines multimodal conversational systems and Augmentative and Alternative Communication (AAC)-oriented chatbots. Alex, a multimodal communication tool combining symbols and speech synthesis, provides non-verbal children with an accessible and customizable interaction space (6). Research on AAC and simplified linguistic structures similarly highlights the value of reduced ambiguity and concise phrasing for autistic users (21).

### 2.8 Screening and assessment applications

Conversational interaction has also been explored in diagnostic and pre-screening contexts. Aquabot is one of the few chatbots specifically designed for autism screening: using natural language processing and decision-tree classification, it assigns severity levels based on user responses and achieved 88% accuracy compared to clinician assessment (19). These results illustrate the potential role of conversational agents in structured diagnostic pre-assessment. Finally, linguistic and interaction-analytic studies provide essential insights for the design of conversational agents. Research examining autistic conversational patterns shows tendencies toward rigidity, difficulties in conversational repair, and atypical timing, emphasising the need for clarity, pacing, and reduced ambiguity in dialogic design (22).

Taken together, these eight macro-areas demonstrate how conversational technologies can create stable and adaptive environments in which autistic individuals may explore communication, rehearse linguistic or emotional skills, and engage in structured interaction at their own pace. Yet these principles remain scattered across isolated applications, without integration into a unified framework that connects conversational interaction to autonomy and functional cognition. By highlighting recurring principles—predictability, personalization, low social pressure, and multimodal support—this body of work provides the conceptual foundations for transitioning from isolated applications to a unified approach, paving the way for the NCF presented in the next section. Table 1 summarizes the eight macro-areas and provides representative (non-exhaustive) systems and empirical contributions for each category.

## 3 The NCF

The NCF builds on the recurring principles identified across existing conversational systems—predictability, personalization, and low social pressure—to propose a unified conceptual foundation for designing chatbots that support autonomy and functional cognition in individuals on the autism spectrum. The framework draws on mediated learning theory and executive functioning research, conceptualizing conversational interaction as a form of cognitive scaffolding. Importantly, the NCF adopts a socio-cognitive view of conversational technologies: dialogue is not treated as “communication vs. cognition,” but as the mechanism through which cognitive scaffolding and functional learning can be delivered in an accessible, low-demand format. Rather than conceiving conversational agents solely as communication tools, the NCF frames them as structured and adaptive learning environments in which dialogue becomes a vehicle for practicing planning, organization, and goal-directed behavior. At the core of the framework lies the assumption that conversational interaction can function as a form of guided cognitive exercise when characterized by clear prompts, predictable turn-taking, and a gradual modulation of task complexity. Within this perspective, the chatbot acts as a cognitive support tool, providing scaffolding that helps users sequence actions, monitor progress, and reduce reliance on external cues—while maintaining a complementary role to human guidance rather than replacing it. The NCF is articulated along three main axes, summarized in Table 2.

**Table 2:** Core dimensions of the NCF and their expected impact on learning and autonomy.

Axis	Core focus	Key principles	Expected outcome
Cognitive–functional	Support for autonomy through structured conversational learning	Reinforcement of organization, sequencing, and problem-solving strategies	Improved functional independence and transfer of skills to daily contexts
Structural–adaptive	Balance between predictability and flexibility	Progressive calibration of language, pace, and task complexity	Cognitive security combined with sustained engagement
Contextual–ecological	Integration of learning into real-life situations	Use of conversational routines and ecological contexts	Generalization of learned abilities and long-term maintenance

### 3.1 Cognitive–functional axis

The cognitive–functional axis highlights conversational activities that reinforce task management, sequencing, and everyday problem-solving, thereby engaging foundational executive components relevant to daily functioning. For example, a chatbot may guide the user through the routine “getting the bag ready before leaving home,” breaking the task into predictable, trackable micro-steps that support sequencing and action monitoring.

### 3.2 Structural–adaptive axis

The structural–adaptive axis emphasizes the balance between stability and flexibility, ensuring that conversational pacing, linguistic complexity, and difficulty levels adjust to user responses without compromising predictability. For instance, when the system detects unusually short responses, hesitation, or slowed interaction, it can automatically simplify its language, lengthen pauses, or reduce multitasking demands to prevent cognitive overload. Crucially, such adaptations must remain predictable; abrupt changes in pacing or linguistic complexity may compromise cognitive security for autistic users.

### 3.3 Contextual–ecological axis

Finally, the contextual–ecological axis focuses on embedding conversational practice within meaningful real-life situations, promoting the generalization and maintenance of acquired skills beyond the digital environment. For example, during meal preparation time, the chatbot may activate a context-linked conversational routine offering step-by-step prompts aligned with the user's environment—such as the time of day or location—to support action initiation and ecological relevance.

Overall, this socio-cognitive framing guides chatbot design toward predictable, autonomy-oriented routines rather than social simulation as an end in itself. Its purpose is to guide the development of conversational systems that strengthen everyday independence, support functional learning, and foster more sustainable forms of autonomy over time. Positioned within existing autonomy-oriented assistive technology traditions, the NCF synthesizes scaffolding and cognitive-support principles for conversational agents and articulates a set of design axes linking dialogue structure to functional cognition. Finally, the NCF is not intended to replace human intervention, nor should it function as a standalone tool. Its validity depends on integration with human guidance and empirical evaluation of its impact on functional cognition.

## 4 Future perspectives

Drawing on the evidence summarized above, future work on conversational technologies for autism may be strengthened by more explicit conceptual and empirical links between conversational design choices, underlying cognitive mechanisms, and real-world autonomy outcomes. More specifically, progress will benefit from connecting interaction features such as prompt structure, turn-taking, pacing, and predictability to targeted cognitive processes, including planning, sequencing, initiation, and monitoring, and then to functional outcomes, particularly generalization to daily routines. For clarity, we outline four interconnected directions: cognitive empowerment and empirical testing; biofeedback-informed adaptivity; neuroinclusive interface and interaction design; and contextual, routine-based deployment to support generalization. Within this perspective, a chatbot is not merely a communication tool, but a structured environment in which individuals can practice planning, organization, and routine management through clear and progressively structured interactions. Crucially, these benefits emerge through socio-cognitive dialogue: communicative interaction is the mechanism through which cognitive scaffolding can be delivered, rather than a separate or competing function. Bringing together functional and cognitive dimensions in this way represents, in our view, one of the most promising directions for designing tools that are genuinely useful and transferable across everyday contexts.

### 4.1 Cognitive empowerment and empirical testing

A priority for future research is to operationalize cognitive empowerment in measurable and falsifiable ways, moving beyond broad claims of engagement toward outcomes tied to functional cognition. Relevant endpoints include task initiation, step completion, error recovery, prospective remembering, and routine adherence. In this sense, the NCF can serve as a basis for experimental studies testing how specific conversational design elements—such as predictable prompt templates, graded task complexity, and explicit monitoring prompts—shape functional and cognitive outcomes in autistic users. This direction also requires stronger methodological consistency, including clearly defined outcome measures, ecologically valid tasks, and follow-up assessments of maintenance and generalization.

### 4.2 Biofeedback-informed adaptivity

A second area of advancement concerns the integration of biofeedback, which we consider essential for making conversational interactions more responsive to the user's internal state. Research on physiological monitoring—such as heart rate variability and electrodermal activity—shows that these signals can capture moments of overload or shifts in emotional regulation (23). While promising, the integration of physiological monitoring in autism remains preliminary and requires strong safeguards related to privacy, sensory tolerance, and interpretability (24). Integrating biofeedback into conversational design is not a mere technological enhancement but a fundamental requirement for ensuring cognitive and sensory accessibility. Physiological indicators can inform the conversational agent about the user's cognitive load, emotional arousal, or sensory thresholds, allowing the system to adapt the interaction in real time—for instance by simplifying linguistic demands, reducing task load, or increasing the predictability of prompts (25). In this sense, biofeedback serves as a bridge between internal regulation processes and external conversational support, enabling the chatbot to anticipate and prevent overload rather than reacting only after engagement has diminished (26). Future implementations should also address data governance explicitly—what is collected, where it is stored, and who can access it—and prioritize transparent, user-centered control over adaptive features.

### 4.3 Neuroinclusive interface and interaction design

We also view interface design as a central element for future innovation. Evidence from studies on perceptual processing in autism indicates that low-saturation color palettes, moderated contrast levels, and visually regular layouts can reduce sensory load and support sustained attention (27, 28, 32). Similarly, research on visual complexity shows that simplified layouts, clear information hierarchies, and coherent graphical structures improve comprehension and task performance (32). These principles are consistent with Universal Design for Learning (UDL) and visual scaffolding approaches, which further support comprehension and reduce cognitive load. Findings from Augmentative and Alternative Communication further emphasize the value of highly iconic symbols and predictable visual transitions, which facilitate information decoding and reduce cognitive load for users with linguistic or pragmatic challenges (21). Incorporating these design principles into chatbot interfaces is, in our view, essential for creating digital environments that are perceptually accessible and cognitively sustainable. Beyond visual layout, “interaction design” should be treated as part of neuroinclusion: predictable turn-taking, stable prompt formats, and explicit repair strategies can reduce ambiguity and support sustained participation over time.

### 4.4 Contextual, routine-based interaction and ecological validity

Alongside interface design, multimodal and context-sensitive interaction represents a further trajectory for development. Combining conversational guidance with structured visual supports and contextual cues—such as location, time of day, or ongoing activity—can strengthen the connection between digital prompts and real-world actions. Research on technology use in autism underscores the importance of ecological relevance for skill generalization (29). A chatbot capable of integrating contextual information may therefore function as an effective facilitator of autonomy, offering guidance that is both actionable and situated in the user's real environment. This direction should explicitly target generalization mechanisms, for example cueing routines *in situ*, fading prompts over time, and supporting transfer across settings, rather than assuming that improvements during interaction will automatically translate to daily functioning. Taken together, these directions point toward a vision of conversational technology that prioritizes personalization, accessibility, and ecological validity rather than technological sophistication for its own sake (30). Equally essential is the adoption of participatory and neurodivergent-led design practices, ensuring that autistic users actively shape the conversational tools intended to support them. Finally, as these systems become more autonomous and adaptive, interdisciplinary integration spanning clinical expertise, HCI, cognitive science, ethics, and data privacy will be necessary to ensure safety, interpretability, and sustained real-world usefulness. By integrating biofeedback, neuroinclusive interface design, and context-sensitive adaptivity, future chatbots may evolve into stable cognitive environments that support the acquisition and consolidation of functional skills. These systems are not intended to replace human guidance, but to complement it, supporting autonomy that is sustainable, transferable, and responsive to the individual needs of autistic users.

## 5 Conclusion

In conclusion, conversational technologies show increasing potential in supporting aspects of daily life that can be challenging for many autistic individuals. Available evidence indicates that, when designed with principles of predictability, personalization, and cognitive accessibility, chatbots can create structured environments that complement human guidance and promote more effective engagement in everyday activities. The NCF offers a direction for integrating these principles into coherent design strategies that connect conversational structure with the user's cognitive and functional needs. Interdisciplinary contributions will be essential to translate these insights into concrete tools that can be applied across real-life settings. Rather than framing conversational agents as cognitive scaffolds instead of social simulators, the NCF adopts a socio-cognitive perspective in which structured dialogue can support functional autonomy precisely through interaction—integrating communicative and cognitive dimensions toward everyday goals. Accordingly, the NCF does not claim an entirely new direction for assistive technology, but offers a chatbot-specific synthesis that organizes autonomy-oriented support principles into actionable design axes linking dialogue structure, cognitive mechanisms, and functional outcomes. We contend that the future of assistive conversational technologies depends on embracing neuroinclusive principles as core design requirements, ensuring that such tools genuinely expand opportunities for autonomy and participation. Ultimately, the value of conversational systems does not lie in replacing human relationships, but in expanding opportunities for autonomy, participation, and learning in daily life. Meaningful progress in this field will require active involvement of autistic individuals across all design and evaluation phases and rigorous empirical validation of functional and cognitive outcomes in ecologically meaningful contexts.

## Data Availability

The original contributions presented in the study are included in the article/Supplementary Material, further inquiries can be directed to the corresponding author.
